# Diversity of ligninolytic ascomycete fungi associated with the bleached leaf litter in subtropical and temperate forests

**DOI:** 10.3934/microbiol.2024042

**Published:** 2024-11-15

**Authors:** Momoka Yoneda, Hiroki Ameno, Ayaka Nishimura, Kohei Tabuchi, Yuki Hatano, Takashi Osono

**Affiliations:** 1 Graduate School of Science and Engineering, Doshisha University, Kyotanabe, Kyoto 610-0394, Japan; 2 Faculty of Science and Engineering, Doshisha University, Kyotanabe, Kyoto 610-0394, Japan

**Keywords:** Ascomycota, dead leaves, fungal richness, Hypoxylaceae, lignin decomposition, Xylariaceae

## Abstract

Little is known regarding the diversity patterns of Xylariaceae and Hypoxylaceae (Ascomycota) fungi taking part in the lignin decomposition of leaf litter from different tree species and under different climatic regions. The alpha and beta diversity of Xylariaceae and Hypoxylaceae fungi was investigated on bleached leaf litter from nine subtropical and cool temperate tree species in Japan. A total of 248 fungal isolates, obtained from 480 leaves from the nine tree species, were classified into 43 operational taxonomic units (OTUs) with a 97% similarity threshold and were assigned to nine genera of Xylariaceae and Hypoxylaceae. There was no overlap of fungal OTUs between subtropical and cool temperate trees. The mean number of fungal OTUs was generally higher in subtropical than cool temperate trees, whereas rarefaction curves depicting the numbers of OTU with respect to the number of leaves from which fungi were isolated were less steep in subtropical trees than in cool temperate trees, reflecting the dominance of major OTUs in the subtropical trees and indicating a higher species richness in cool temperate regions. Nonmetric multidimensional scaling showed general overlaps of fungal OTU compositions among tree species in the respective climatic regions, and one-way permutational multivariate analysis of variance indicated that the OTU composition was not significantly different between the tree species. These results suggest a wide host range and some geographic and climatic structures of distribution of these ligninolytic fungi.

## 1. Introduction

Fungi are eukaryotic microorganisms that serve as a major component of soil biodiversity and play crucial roles in the functioning of soil as decomposers [1]. Fungi are unique in that they are capable of actively attacking lignin in dead plant residues resistant to microbial degradation [2],[3]. The selective decomposition of lignin by fungi results in the white rot of wood and the bleaching of leaf litter [4],[5]. Previous studies on the diversity and ecology of ligninolytic fungi have paid special attention to the Basidiomycota occurring on wood [6], whereas information about the Ascomycota colonizing leaf litter is scarce, even though Xylariaceae and Hypoxylaceae fungi from the *Ascomycota* group are well-known for their ligninolytic activity [7],[8]. Also, recent studies often encountered ascomycete fungi on bleached portions of leaf litter in subtropical [9] and temperate [10] regions. However, there is a lack of knowledge regarding the diversity patterns of Xylariaceae and Hypoxylaceae fungi on leaf litter from different tree species and between climatic regions.

The purpose of the present study was to investigate the alpha and beta diversity of Xylariaceae and Hypoxylaceae fungi undertaking lignin decomposition from leaf litter of subtropical and cool temperate tree species in Japan. In this context, we selected nine tree species that are major forest components in these climatic regions and isolated fungi responsible for the bleaching of leaf litter of these tree species. The dataset was then analyzed using rarefaction analysis, nonmetric multidimensional scaling, and one-way permutational multivariate analysis of variance to test the hypothesis that fungal alpha and beta diversity were different among tree species and between subtropical and cool temperate regions.

## 2. Materials and methods

### 2.1. Study sites and sample collection

Samples were collected from two study sites in Japan—a subtropical forest in Yona, Okinawa (26°44′17″–32″N, 128°13′60″–14′9″E, 250–330 m a.s.l.) and a cool temperate forest in Sanada, Nagano (36°31′5″–36°40′24″N, 138°13′7″–21′11″E, 1280–1340 m a.s.l.) (Figure S1). Mean annual temperature of the subtropical and cool temperate forests was 21.8 °C and 6.9 °C, respectively, and mean annual precipitation was 2680 mm and 1200 mm, respectively. The subtropical forest is located in the northern part of Okinawa Island in southern Japan and is dominated by *Castanopsis sieboldii* (Makino) Hatusima (*Fagaceae*) and *Schima wallichii* (DC.) Korthals (*Theaceae*) [11]. Seven evergreen broad-leaved tree species from six families were selected (Table 1) as these are major or dominant components of the forest. Sampling was conducted once in September 2020 at four forest stands around the Yona Experimental Forest of the University of the Ryukyus. The cool temperate forest is located in the central part of Honshu Island in central Japan and is dominated by the deciduous broad-leaved tree species *Fagus crenata* Bl. and *Quercus crispula* Bl. (*Fagaceae*). These two dominant tree species were selected (Table 1), and sampling was conducted once in August 2020 at four forest stands in and around the Sugadaira Montane Research Center of University of Tsukuba. The quadrats (5 × 5 m) were arbitrarily selected beneath the tree canopy and at least 30 m from each other. Sampling of 10 bleached leaves was done per quadrat, with 3–9 quadrats per tree species, in a total of 7 and 2 tree species in the subtropical and cool temperate forest for a total of 320 and 160 leaves, respectively (Table 1). Leaf litter with evident bleaching on the leaf surface and in which the original leaf area was preserved were collected from the surface of the forest floor within the quadrats. Leaves were placed in paper bags, preserved at room temperature (approximately 15–20 °C), and taken back to the laboratory.

### 2.2. Fungal isolation

Single leaf disks were excised from the bleached portion of individual leaves using a sterile 6 mm diameter cork borer. One leaf disk was excised per leaf, producing a total of 480 leaf disks of bleached portions for the isolation of fungi. Fungi were isolated from the disks using the surface disinfection method [12] using modified lignocellulose agar. Putative Xylariaceae and Hypoxylaceae fungi that produced anamorphic conidia and conidiophores on the plates, such as *Xylocoremium*, *Geniculosporium*, and *Nodulisporium*, and/or dark pseudosclerotinial plates in submerged hyphae were subcultured to establish pure cultures. These isolates were then tested for their potential ability to bleach leaf litter under a pure culture condition, according to the method described previously [9], using sterilized newly shed leaves of *C. sieboldii* for subtropical isolates and *F. crenata* for cool temperate ones. Fungal isolates that caused the bleaching of sterilized leaves under the pure culture condition were regarded as ligninolytic fungi and analyzed further.

### 2.3. Determination of OTUs and taxonomic assignment

The rDNA ITS sequences of the ligninolytic fungal isolates were analyzed, following methods described previously [9] and using primer pairs of ITS1F and LR3. The ITS sequences that originated from isolates were grouped into operational taxonomic units (OTUs) according to the 97% similarity criterion and were then assembled using VSEARCH as implemented in Claident pipeline v0.2.2018.05.29 (Table S1) (software available online: https://www.claident.org/). For each of the OTUs, taxonomic identification was conducted based on the query-centric auto-k-nearest neighbor (QCauto) method [13] with the reference database from International Nucleotide Sequence Database Collaboration (INSDC) and subsequent taxonomic assignment with the lowest common ancestor (LCA) algorithm as implemented in Claident. The ITS sequences were also compared with the rDNA sequences available in the GenBank database by means of BLAST+ [14]. The ITS sequences determined in the present study were deposited in the DNA Data Bank of Japan (DDBJ) (LC821383–LC821630) (Table S2). We retrieved one fungal isolate of each OTU per leaf disk even when two or more isolates of the same OTU appeared.

**Table 1.** Location and climatic region of study sites, tree species examined, and the summarized results of isolation of Xylariaceae and Hypoxylaceae fungi. Values indicate mean ± standard error. The same letters indicate that there is not a significant difference at the 5% level by Tukey's HSD test.

Location (Climate)	Tree species	Plant family	Code	Number of leaves examined		Number of leaves isolated	Total number of fungal OTUs	Mean number of fungal OTUs
Yona, Okinawa	*Castanopsis sieboldii*	*Fagaceae*	Cs	60	41	(68.3%)	9	0.68 ± 0.10 ab
(Subtropical)	*Schima wallichii*	*Theaceae*	Sw	60	29	(48.3%)	9	0.48 ± 0.08 abc
	*Neolitsea sericea*	*Lauraceae*	Ns	50	25	(50.0%)	9	0.50 ± 0.09 abc
	*Machilus thunbergii*	*Lauraceae*	Mt	40	23	(57.5%)	6	0.58 ± 0.11 abc
	*Elaeocarpus japonicus*	*Elaeocarpaceae*	Ej	40	34	(85.0%)	11	0.85 ± 0.12 a
	*Distylium racemosum*	*Hamamelidaceae*	Dr	40	28	(70.0%)	10	0.70 ± 0.13 ab
	*Myrica rubra*	*Myricaceae*	Mr	30	21	(70.0%)	4	0.70 ± 0.12 ab
Sanada, Nagano	*Fagus crenata*	*Fagaceae*	Fc	90	31	(34.4%)	13	0.34 ± 0.06 bc
(Cool temperate)	*Quercus crispula*	*Fagaceae*	Qc	70	16	(22.9%)	11	0.23 ± 0.05 c

### 2.4. Data reduction and statistical analyses

A datasheet was constructed showing the presence/absence of individual fungal OTUs on the 480 leaf disks from the nine tree species. The frequency of occurrence of each OTU was then calculated for individual tree species as a percentage of the number of leaf disks from which that particular OTU was isolated with respect to the total number of leaf disks examined (i.e., 30–90). A generalized linear model (GLM) was used to examine the effects of tree species on the number of fungal OTUs. The glm function of R software was used to perform the analysis, with the glht function of the ‘multcomp’ package for multiple comparisons with Tukey's test. Rarefaction analysis was then conducted to compare the number of fungal OTUs between the tree species by adjusting the difference in the number of fungal isolates across tree species, using the specaccum function with the method ‘coleman’ of the vegan package of R software [15]. Next, nonmetric multidimensional scaling (NMDS) with the Jaccard distance metric was used to analyze the variation of fungal OTU composition between tree species. The NMDS analysis was carried out with the metaNDS function with the default settings of the vegan package. One-way permutational multivariate analysis of variance (PERMANOVA) was then conducted to test the effect of tree species on the dissimilarity of OTU composition. Data of singleton OTUs were excluded from the dataset when performing NMDS and PERMANOVA. Moreover, datasets of subtropical and cool temperate trees were analyzed separately in NMDS and PERMANOVA as there was no overlap of fungal OTUs between these regions. All analyses were performed using RStudio for Macintosh version 1.4.1717.

## 3. Results

### 3.1. Taxonomic assignment

A total of 248 fungal isolates were obtained from 202 (42.1%) of the 480 leaf disks examined for nine tree species, with 16–41 isolates per tree species (Table 1). The frequency at which fungi were isolated in leaves was generally higher in subtropical tree species (48.3%–85.0%) than in cool temperate tree species (22.9%–34.4%). The 248 fungal isolates were classified into 43 OTUs at the 97% similarity threshold and were assigned to Xylariaceae (36 OTUs) and Hypoxylaceae (7 OTUs) (Table 2; Figure S2). Claident assigned these OTUs to nine genera, with the most OTU-rich genus being *Xylaria* (Xylariaceae, 18 OTUs), followed by *Nemania* (Xylariaceae, 11 OTUs), *Hypoxylon* (Hypoxylaceae, 5 OTUs), *Biscogniauxia* (Xylariaceae, 3 OTUs), *Annulohypoxylon* and *Daldinia* (Hypoxylaceae, 1 OTU each), and *Nodulisporium*, *Rosellinia*, and *Whalleya* (Xylariaceae, 1 OTU each). The remaining OTU belonged to Xylariaceae but was not assigned to a genus. There was no overlap of fungal OTUs between subtropical (24 OTUs) and cool temperate tree species (19 OTUs), and all seven Hypoxylaceae OTUs were isolated from cool temperate trees (Table 2). The most abundant OTU was *Xylaria cubensis* OTU_20 isolated from seven subtropical leaves, accounting for 116 (46.8%) of the 248 isolates, followed by *Nemania diffusa* OTU_03 (14 isolates, 5.6%) from cool temperate leaves and *N. diffusa* OTU_21, *Xylaria* sp. OTU_24, and *X. cubensis* OTU_27 (13 isolates each, 5.2%) from subtropical leaves. Twenty-six (61.0%) of the 43 OTUs were detected only once and were singletons (Table 2), with 12 and 14 being from cool temperate and subtropical trees, respectively.

### 3.2. OTU richness and composition

**Figure 1.**
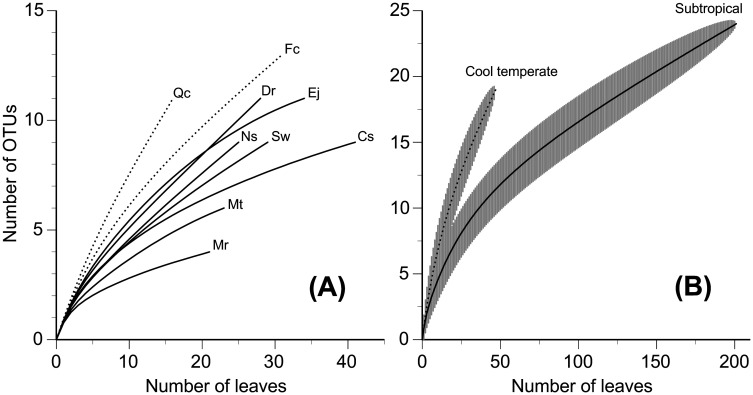
Rarefaction curve depicting the effect of the number of leaves examined on the number of fungal OTUs. Solid line, subtropical tree species; dotted line, cool temperate tree species. (A) Data for nine tree species. Abbreviated codes of tree species are as in Table 1. Standard deviations are not shown for the sake of visibility. (B) Combined data for all subtropical species and combined data for all cool temperate tree species are shown. Shaded areas indicate standard deviations of the means.

The total number of fungal OTUs per tree species ranged from 4 to 13, and the mean number of fungal OTUs was significantly higher in *Elaeocarpus japonicus* and other subtropical tree species than in *Quercus crispula* and *Fagus crenata* from the cool temperate region (Table 1). Rarefaction curves depicting the numbers of OTU with respect to the number of leaves from which fungi were isolated indicated that, in the present study, the OTU richness of the nine tree species did not reach asymptotes (Figure 1). This tendency was primarily attributed to the relatively high number of singleton OTUs (Table 2). The rarefaction curve was generally less steep in the seven subtropical trees than in the two cool temperate trees (Figure 1A), due to the dominance (i.e., relatively high frequency of occurrence) of *X. cubensis* OTU_20 in the subtropical trees and the greater proportions of singleton OTUs in the cool temperate trees. In addition, we combined the data for subtropical and cool temperate tree species to construct two datasets and two rarefaction curves for these climatic regions, because the OTU compositions were not significantly different among leaves of the tree species from the respective climatic regions (see below). The rarefaction curve was again less steep in the subtropical than in the cool temperate dataset (Figure 1B), reflecting the dominance of major OTUs in the subtropical trees and indicating the greater species richness in cool temperate regions.

**Figure 2.**
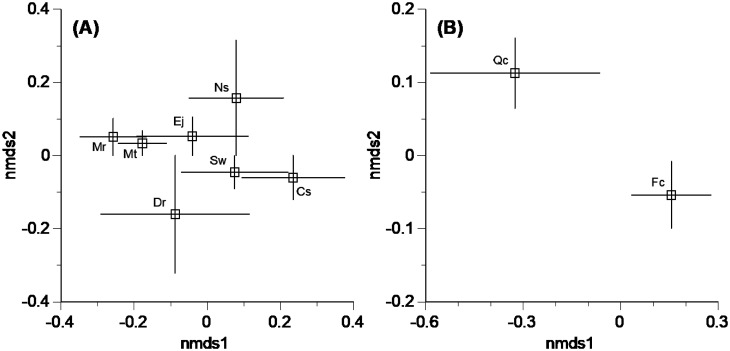
Multivariate ordination of fungal OTU composition in leaf litter of subtropical (A) and cool temperate tree species (B), using nonmetric multidimensional scaling (NMDS, stress value = 0.016 in subtropical dataset and 0.000 in cool temperate dataset). Abbreviated codes of tree species are as in Table 1. Bars indicate standard errors.

Dissimilarity of OTU composition was ordinated using NMDS separately for seven subtropical and two cool temperate trees, resulting in wide variations within single tree species and general overlaps among tree species in the respective climatic regions (Figure 2). In the PERMANOVA, the OTU composition was not significantly different between tree species both for subtropical (F = 1.2918, p = 0.143) and cool temperate datasets (F = 2.1909, p = 0.055).

**Table 2.** Taxon and frequency of occurrence of 43 fungal OTUs.

OTU	Taxon	Family	Subtropical							Cool temperate	
			Cs	Mr	Sw	Ej	Ns	Mt	Dr	Fc	Qc
OTU_20	*Xylaria cubensis*	*Xylariaceae*	36.7	53.3	26.7	40.0	30.0	40.0	27.5	0.0	0.0
OTU_21	*Nemania diffusa*	*Xylariaceae*	10.0	0.0	6.7	5.0	0.0	0.0	0.0	0.0	0.0
OTU_22	*Nemania bipapillata*	*Xylariaceae*	8.3	0.0	0.0	5.0	6.0	0.0	0.0	0.0	0.0
OTU_23	*Xylaria* sp.	*Xylariaceae*	1.7	0.0	0.0	0.0	0.0	0.0	0.0	0.0	0.0
OTU_24	*Xylaria* sp.	*Xylariaceae*	3.3	10.0	3.3	5.0	2.0	5.0	2.5	0.0	0.0
OTU_25	*Xylaria* sp.	*Xylariaceae*	1.7	0.0	0.0	0.0	0.0	0.0	2.5	0.0	0.0
OTU_26	*Xylaria* sp.	*Xylariaceae*	3.3	0.0	1.7	2.5	0.0	0.0	0.0	0.0	0.0
OTU_27	*Xylaria cubensis*	*Xylariaceae*	1.7	3.3	1.7	10.0	0.0	2.5	12.5	0.0	0.0
OTU_28	*Nodulisporium* sp.	*Xylariaceae*	1.7	0.0	1.7	2.5	0.0	0.0	2.5	0.0	0.0
OTU_29	*Xylaria* sp.	*Xylariaceae*	0.0	3.3	1.7	5.0	0.0	2.5	2.5	0.0	0.0
OTU_30	*Xylaria cubensis*	*Xylariaceae*	0.0	0.0	1.7	5.0	2.0	5.0	0.0	0.0	0.0
OTU_31	*Nemania diffusa*	*Xylariaceae*	0.0	0.0	1.7	0.0	0.0	0.0	0.0	0.0	0.0
OTU_32	*Biscogniauxia petrensis*	*Xylariaceae*	0.0	0.0	0.0	2.5	0.0	0.0	0.0	0.0	0.0
OTU_33	*Nemania abortiva*	*Xylariaceae*	0.0	0.0	0.0	2.5	0.0	0.0	0.0	0.0	0.0
OTU_34	*Xylaria* sp.	*Xylariaceae*	0.0	0.0	0.0	0.0	2.0	0.0	0.0	0.0	0.0
OTU_35	*Xylaria cubensis*	*Xylariaceae*	0.0	0.0	0.0	0.0	2.0	0.0	0.0	0.0	0.0
OTU_36	*Xylaria cubensis*	*Xylariaceae*	0.0	0.0	0.0	0.0	2.0	0.0	0.0	0.0	0.0
OTU_37	*Nemania bipapillata*	*Xylariaceae*	0.0	0.0	0.0	0.0	2.0	0.0	0.0	0.0	0.0
OTU_38	*Xylaria* sp.	*Xylariaceae*	0.0	0.0	0.0	0.0	2.0	0.0	0.0	0.0	0.0
OTU_39	*Xylaria cubensis*	*Xylariaceae*	0.0	0.0	0.0	0.0	0.0	2.5	0.0	0.0	0.0
OTU_40	*Xylariaceae* sp.	*Xylariaceae*	0.0	0.0	0.0	0.0	0.0	0.0	2.5	0.0	0.0
OTU_41	*Xylaria* sp.	*Xylariaceae*	0.0	0.0	0.0	0.0	0.0	0.0	2.5	0.0	0.0
OTU_42	*Xylaria cubensis*	*Xylariaceae*	0.0	0.0	0.0	0.0	0.0	0.0	2.5	0.0	0.0
OTU_43	*Nemania diffusa*	*Xylariaceae*	0.0	0.0	0.0	0.0	0.0	0.0	2.5	0.0	0.0
OTU_01	*Annulohypoxylon* sp.	*Hypoxylaceae*	0.0	0.0	0.0	0.0	0.0	0.0	0.0	1.1	0.0
OTU_02	*Xylaria carpophila*	*Xylariaceae*	0.0	0.0	0.0	0.0	0.0	0.0	0.0	5.6	0.0
OTU_03	*Nemania diffusa*	*Xylariaceae*	0.0	0.0	0.0	0.0	0.0	0.0	0.0	12.2	4.3
OTU_04	*Hypoxylon* cf. *rubiginosum*	*Hypoxylaceae*	0.0	0.0	0.0	0.0	0.0	0.0	0.0	1.1	1.4
OTU_05	*Whalleya microplaca*	*Xylariaceae*	0.0	0.0	0.0	0.0	0.0	0.0	0.0	1.1	1.4
OTU_06	*Nemania* sp.	*Xylariaceae*	0.0	0.0	0.0	0.0	0.0	0.0	0.0	3.3	1.4
OTU_07	*Hypoxylon perforatum*	*Hypoxylaceae*	0.0	0.0	0.0	0.0	0.0	0.0	0.0	3.3	1.4
OTU_08	*Hypoxylon cercidicola*	*Hypoxylaceae*	0.0	0.0	0.0	0.0	0.0	0.0	0.0	1.1	0.0
OTU_09	*Hypoxylon ferrugineum*	*Hypoxylaceae*	0.0	0.0	0.0	0.0	0.0	0.0	0.0	1.1	0.0
OTU_10	*Nemania* sp.	*Xylariaceae*	0.0	0.0	0.0	0.0	0.0	0.0	0.0	1.1	0.0
OTU_11	*Xylaria cubensis*	*Xylariaceae*	0.0	0.0	0.0	0.0	0.0	0.0	0.0	1.1	0.0
OTU_12	*Nemania diffusa*	*Xylariaceae*	0.0	0.0	0.0	0.0	0.0	0.0	0.0	1.1	0.0
OTU_13	*Hypoxylon* sp.	*Hypoxylaceae*	0.0	0.0	0.0	0.0	0.0	0.0	0.0	1.1	0.0
OTU_14	*Biscogniauxia maritima*	*Xylariaceae*	0.0	0.0	0.0	0.0	0.0	0.0	0.0	0.0	5.7
OTU_15	*Daldinia childiae*	*Hypoxylaceae*	0.0	0.0	0.0	0.0	0.0	0.0	0.0	0.0	1.4
OTU_16	*Nemania* sp.	*Xylariaceae*	0.0	0.0	0.0	0.0	0.0	0.0	0.0	0.0	1.4
OTU_17	*Nemania serpens*	*Xylariaceae*	0.0	0.0	0.0	0.0	0.0	0.0	0.0	0.0	1.4
OTU_18	*Rosellinia aquila*	*Xylariaceae*	0.0	0.0	0.0	0.0	0.0	0.0	0.0	0.0	1.4
OTU_19	*Biscogniauxia maritima*	*Xylariaceae*	0.0	0.0	0.0	0.0	0.0	0.0	0.0	0.0	1.4

## 4. Discussion

The present study estimated the alpha diversity (i.e., OTU richness) and beta diversity (OTU composition) of ligninolytic fungi from Xylariaceae and Hypoxylaceae associated with bleached leaf litter from subtropical and temperate forest soils. The eight fungal genera found (*Xylaria*, *Nemania*, *Hypoxylon*, *Biscogniauxia*, *Annulohypoxylon*, *Daldinia*, *Nodulisporium*, and *Rosellinia*) have been isolated previously from bleached leaf litter in both subtropical and temperate forests [9],[10]. Xylariaceae and Hypoxylaceae are closely related taxa of the Xylariales and are well-known for their ability to decompose lignin in plant residues [16]–[18]. Wendt et al. [19] recently re-established Hypoxylaceae (formerly classified as subfamily Hypoxyloideae in Xylariaceae) based on morphology, phylogeny, and secondary metabolite analyses. According to the survey carried out by the authors in main databases such as PubMed, Scopus, and Web of Science, this is the first report of *Whalleya microplaca* OTU_05 as ligninolytic fungi isolated from cool temperate leaves (Table 2). This species was previously known as wood-inhabiting fungi fruiting on dead wood [20] and as endophytes of live leaves [21].

The rarefaction curves (Figure 2) did not reach asymptotes due to the relatively high number of singleton OTUs but suggested a lower alpha diversity of ligninolytic ascomycete fungi in the subtropical than in the cool temperate region. However, caution must be taken to ecologically interpret this climatic difference in fungal diversity because the numbers of sites and tree species examined were low, especially in the cool temperate region. Few comparative data have been available regarding climatic patterns of ligninolytic ascomycete fungi in leaf litter, but a similar study was conducted to compare the diversity of endophytic fungi in Xylariaceae and Hypoxylaceae (as Xylarioideae and Hypoxyloideae of Xylariaceae) in live leaves [22]. Interestingly, Ikeda et al. [22] found an opposite pattern to that found in the present study; namely, they found that the rarefaction curve of endophytic fungi was steeper in the subtropical than in the cool temperate forest, i.e., there was greater diversity of endophytic fungi in the subtropical forest. This discrepancy may be attributable to the difference in substrata (dead vs. live leaves), but more investigations are needed to further consider the climatic pattern of diversity of Xylariaceae and Hypoxylaceae fungi.

Segregation of the OTU composition among tree species was not obvious for either subtropical or cool temperate tree leaves (Figure 2). This is primarily attributable to the dominance of particular fungal OTUs on leaf litter across different tree species, i.e., *X. cubensis* OTU_20 in subtropical trees and *N. diffusa* OTU_03 in cool temperate trees, leading to relatively similar OTU compositions among tree species examined. This result in the present study is consistent with the previous finding [9] that the isolates of *Xylariaceae* obtained from leaf litter had low host recurrence. Zhou and Hyde [23] defined host recurrence of fungi as the frequent or predominant occurrence of a fungus on a particular host or range of hosts, but the fungus can also occur infrequently on other host plants in the same habitat. Indeed, fungi in Xylariaceae and Hypoxylaceae are generally considered to have a wide host range [24]–[26]. In contrast, we found no overlap of fungal OTUs between subtropical and cool temperate tree species (Table 2). Such low similarity in the OTU composition of Xylarioideae and Hypoxyloideae between these climatic regions was previously reported for endophytic fungi [22] and suggests geographic and climatic structures of fungal distribution, at least in Japan. It should be noted, however, that some OTUs from different climatic regions were assigned to the same species, namely *X. cubensis* and *N. diffusa* (Table 2). Such results imply high degrees of intraspecific variability (i.e., more than 3% dissimilarity) in their ITS sequences and/or insufficient taxonomic information in the database record that the bioinformatic tool referred to. In fact, by quantifying the intraspecific ITS variability in all fungi for which sequence data were available through the International Nucleotide Sequence Database (INSD), the intraspecific sequence variability of *X. hypoxylon* was found to reach 24.2% [27].

## 5. Conclusions

The present study demonstrated the patterns of alpha and beta diversity of Xylariaceae and Hypoxylaceae fungi associated with lignin decomposition of leaf litter from subtropical and temperate trees. These fungi are well-known for their ability to metabolize not only lignin but also other diverse compounds [28],[29] and are diverse phylogenetically as well as taxonomically [19],[20]. Future study directions include the evaluation of functional and phylogenetic diversity of Xylariaceae and Hypoxylaceae fungi, in addition to their taxonomic diversity and the climatic patterns of these diversity measures to get further insights into climatic effects on the roles of fungal diversity in forests.

## Supplementary materials

The supporting information can be downloaded at the website.

## Use of AI tools declaration

The authors declare they have not used Artificial Intelligence (AI) tools in the creation of this article.


